# Informing selection of drugs for COVID-19 treatment through adverse events analysis

**DOI:** 10.1038/s41598-021-93500-5

**Published:** 2021-07-07

**Authors:** Wenjing Guo, Bohu Pan, Sugunadevi Sakkiah, Zuowei Ji, Gokhan Yavas, Yanhui Lu, Takashi E. Komatsu, Madhu Lal-Nag, Weida Tong, Tucker A. Patterson, Huixiao Hong

**Affiliations:** 1 National Center for Toxicological Research, U.S. Food and Drug Administration, 3900 NCTR Road, Jefferson, AR 72079 USA; 2 Center for Drug Evaluation and Research, U.S. Food and Drug Administration, 10903 New Hampshire Avenue, Silver Spring, MD 20993 USA

**Keywords:** Drug safety, Data mining, Data processing, Databases

## Abstract

Coronavirus disease 2019 (COVID-19) is an ongoing pandemic and there is an urgent need for safe and effective drugs for COVID-19 treatment. Since developing a new drug is time consuming, many approved or investigational drugs have been repurposed for COVID-19 treatment in clinical trials. Therefore, selection of safe drugs for COVID-19 patients is vital for combating this pandemic. Our goal was to evaluate the safety concerns of drugs by analyzing adverse events reported in post-market surveillance. We collected 296 drugs that have been evaluated in clinical trials for COVID-19 and identified 28,597,464 associated adverse events at the system organ classes (SOCs) level in the FDA adverse events report systems (FAERS). We calculated Z-scores of SOCs that statistically quantify the relative frequency of adverse events of drugs in FAERS to quantitatively measure safety concerns for the drugs. Analyzing the Z-scores revealed that these drugs are associated with different significantly frequent adverse events. Our results suggest that this safety concern metric may serve as a tool to inform selection of drugs with favorable safety profiles for COVID-19 patients in clinical practices. Caution is advised when administering drugs with high Z-scores to patients who are vulnerable to associated adverse events.

## Introduction

Coronavirus disease 2019 (COVID-19), a newly emerged disease caused by severe acute respiratory syndrome coronavirus 2 (SARS-CoV-2), has been rapidly spreading worldwide due to its contagious nature^1–3^. The World Health Organization (WHO) declared COVID-19 a pandemic on March 11, 2020. As of June 3, 2021, the outbreak has been reported in 213 countries with over 171 million confirmed cases and over 3.68 million deaths. The global spread of the virus has overwhelmed healthcare systems and caused unprecedented disruption to society as well as the economy. The severity of this pandemic demands safe and effective therapeutic approaches. Currently, only one drug, remdesivir under the brand name Veklury, is approved by the U.S. Food and Drug Administration (FDA) for adults and pediatric patients (12 years of age and older and weighing at least 40 kg) for the treatment of COVID-19 requiring hospitalization. Since developing a new drug usually takes more than a decade, many COVID-19 studies use an alternative strategy by repurposing approved or investigational drugs to treat COVID-19^4^. Since approved drugs may have been on the market for years or even decades, their safety, toxicity, and pharmacokinetics are known. This knowledge can be used to shorten the time required for developing these drugs to treat COVID-19.

A remarkable number of drugs have been considered for treating COVID-19 patients^5–8^. As of June 3, 2021, 5849 COVID-19-related clinical trials had been registered with ClinicalTrials.gov and 29% (1693) involved the use of marketed drugs. The efficacy and safety of these drugs for treatment of COVID-19-related indications are being tested in ongoing clinical trials. Among these drugs, the antimalarial drugs hydroxychloroquine and chloroquine are widely used in clinical trials. Based on their potential efficacy^9,10^, the FDA and European Medicines Agency (EMA) issued authorization for emergency use of oral formulations of hydroxychloroquine sulfate and chloroquine phosphate in the treatment of COVID-19 patients in late March and early April 2020, respectively. However, on June 15, 2020, the FDA revoked the emergency use authorization (EUA) because these drugs may not be effective in treating COVD-19 and the drugs’ potential benefits for such use do not outweigh their known and potential risks^11,12^. Some drugs emerged in the virtual screening as potential COVID-19 drugs, such as dipyridamole^13^ which inhibits the SARS-CoV-2 main protease, peptides drugs^14^ and chronic disease drugs^15^ including candesartan, losartan, telmisartan. Using the chronic disease drugs as an example, these drugs are in the class of angiotensin II receptor blockers (ARBs) and have shown promising affinities against the COVID-19 main protease in molecular docking and molecular dynamics^15^. Since some clinical trials are still ongoing, the efficacy of those drugs is inconclusive or pending. Therefore, it is important for physicians to select drugs with favorable safety profiles.

Although the efficacy of many drugs in treating COVID-19 patients remains unclear, adverse events reported in post-marketing surveillance can be used as a safety measurement to provide physicians information regarding which adverse events are more likely to occur in patients. This knowledge could be further used to inform selection of drugs for treating COVID-19 patients once drugs are approved/authorized for treatment of specified indications. The FDA adverse events reporting system (FAERS) is a self-reporting adverse events system that is widely used to study the relationship between drugs and adverse events due to the large numbers of adverse event reports in the database^16–18^. In our study, we defined a quantitative safety concern measurement for potential COVID-19 drugs using occurrences of adverse events extracted from FAERS. In brief, we first gathered drugs that have been used to treat COVID-19 in clinical trials and are registered in ClinicalTrials.gov. Then, the adverse events reported for these drugs were retrieved from the FAERS database and were mapped to 27 medical dictionary for regulatory activities (MedDRA) system organ classes (SOCs). We calculated a Z-score for each SOC as a qualitative measure of safety concern to indicate a statistically relative frequency of adverse events in the SOC. This safety concern measurement may help physicians select drugs for treating COVID-19 which are tailored to a patient’s clinical features.

## Results and discussion

### Drugs for COVID-19 treatment in clinical trials

**Figure 1:**
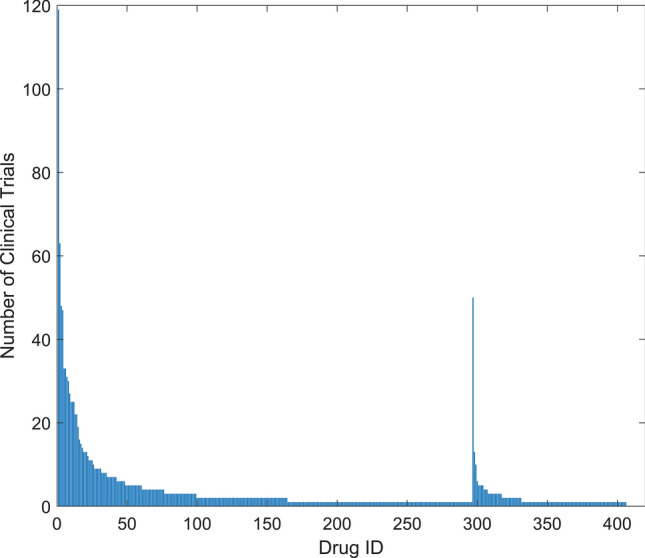
Clinical trials in ClinicalTrials.gov for COVID-19 drugs. The x-axis indicates drug ID that is given in Supplementary Table 1. The y-axis shows the number of clinical trials (data were obtained on June 3, 2021)*.*

To investigate the safety of drugs repurposed for COVID-19, we searched ClinicalTrials.gov on June 3, 2021 and found 5849 clinical trials for COVID-19. To focus on promising COVID-19 drugs, we excluded clinical trials that were marked as withdrawn, suspended, and terminated in the recruitment status and 5599 clinical trials remained. Among these 5599 clinical trials, 1526 were listed as intervention with drugs. Searching the 1526 clinical trials by common drug names and synonyms from the DrugBank database^19^ produced 1075 trials for 406 approved or investigational drugs. The remaining 451 clinical trials without approved or investigational drugs were associated with agents not covered in DrugBank, such as traditional Chinese medicines. These 451 trials were excluded. Detailed information on the clinical trials for the 406 drugs are provided in Supplementary Table 1. Figure 1 shows the distribution of clinical trials involving the 406 drugs. Of these 406 drugs, 207 are being tested in only one clinical trial, 79 in two, 33 in three, and 87 in more than three clinical trials.

Hydroxychloroquine and ivermectin have been utilized in 119 and 63 clinical trials, respectively. It is not surprising that hydroxychloroquine has been extensively studied because it was initially reported to have some inhibitory activity against SARS-CoV-2^12^. The antiparasitic drug, ivermectin, has also been intensively studied for COVID-19 treatment, especially when combined with hydroxychloroquine^8,20^. Other intensively studied drugs include remdesivir with 50 clinical trials, azithromycin with 48 clinical trials, tocilizumab with 47 clinical trials, favipiravir with 33 clinical trials, ritonavir with 33 clinical trials, lopinavir with 31 clinical trials and heparin with 30 clinical trials.

To examine the preferred type of drugs repurposed for COVID-19 treatment, drugs were grouped using the anatomical therapeutic chemical (ATC) classification system. We grouped 237 drugs out of 406 into 14 main pharmacological groups using their first level ATC codes (Supplementary Table 2). The remaining 169 investigational drugs or biological products such as remdesivir and favipiravir were excluded since no ATC codes were assigned to them. All 14 ATC classes are covered by COVID-19 drugs. The antineoplastic and immunomodulating agent class is the largest group with 51 drugs, and blood and blood forming organs is the second largest with 38 drugs. The distribution of clinical trials in the 14 ATC classes is shown in Supplementary Fig. 1. Not surprisingly, the antiparasitic products class contains 239 clinical trials (the most among all 14 classes) due to the large number of clinical trials for hydroxychloroquine (119) and chloroquine (12). The class of anti-infectives for systemic use contains 192 clinical trials for 30 drugs such as azithromycin, lopinavir/ritonavir, umifenovir, and ribavirin. Lopinavir is used with ritonavir to treat HIV-1 infection. Lopinavir/ritonavir’s antiviral effects against SARS-CoV-2 have been reported in some clinical trials and in vitro studies^7,21^. These two drugs have been used as an emergency treatment for COVID-19 patients in many countries including the U.S., Singapore and Japan^7^. However, recent studies have not shown clinical benefit of this combination of drugs for COVID-19^5,22^.

### Adverse events associated with drugs for the treatment of COVID-19

**Figure 2:**
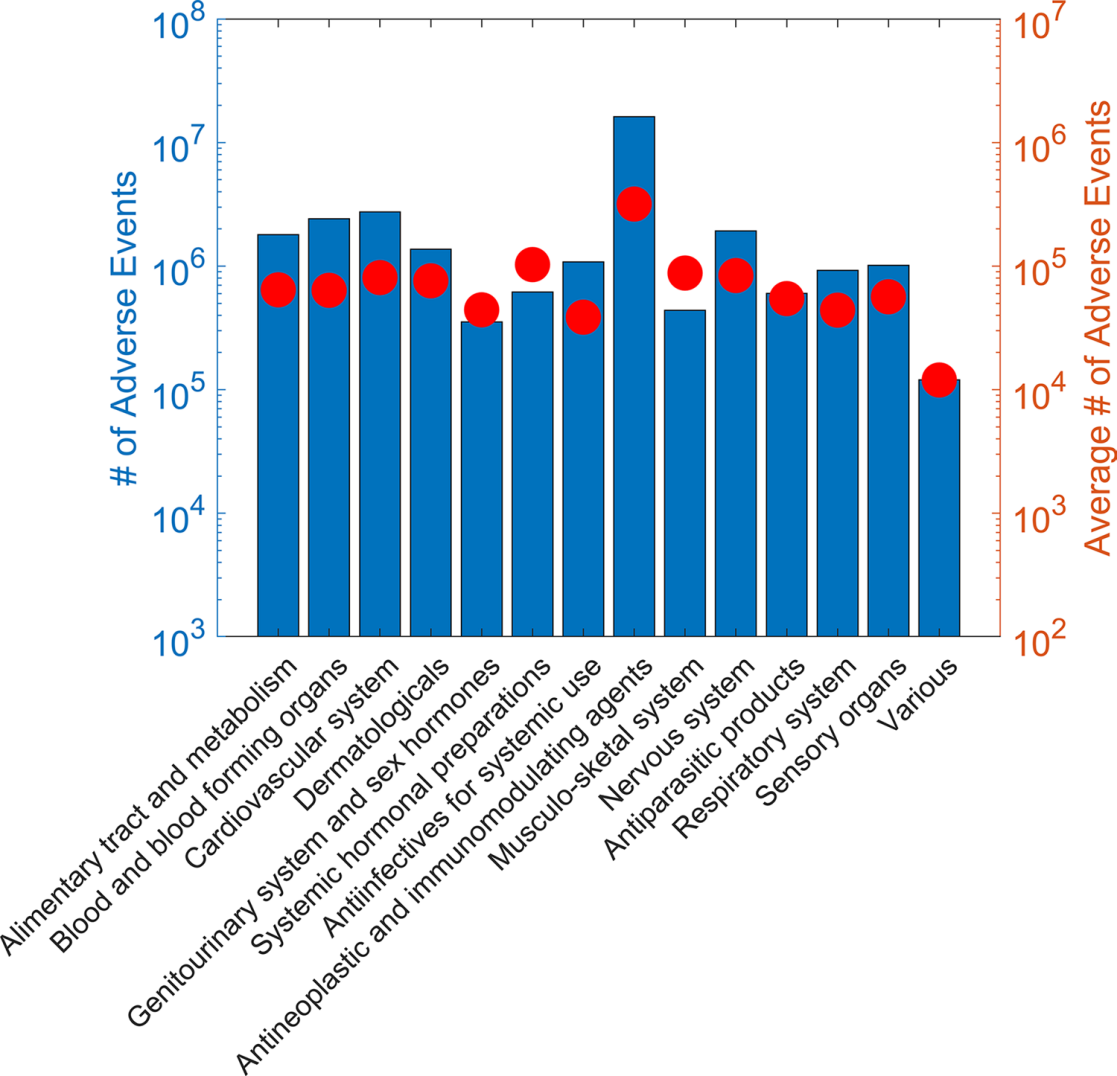
Adverse events in ATC classes. The number of adverse events (left y-axis) were plotted as bars, and the average number of adverse events (right y-axis) were plotted as circles for 14 ATC classes which are depicted on the x-axis.

To measure the safety concerns for drugs that have been used for COVID-19 treatment in clinical trials, we extracted adverse events in post-market surveillance from the FAERS database. Of the 406 drugs found in clinical trials, 296 showed adverse events reported in FAERS; no adverse events were found for the other 110 drugs (Supplementary Table 1). Extracted adverse events coded using MedDRA low level term (LLT) and preferred term (PT) were grouped into 27 SOCs for statistical analysis. The 27 SOCs and their abbreviations used in this study are shown in Supplementary Table 3. All 14 ATC classes of drugs showed adverse events in all 27 SOCs. The number of adverse events for the 14 ATC classes of drugs are provided in Fig. 2. Interestingly, the drugs in the antineoplastic and immunomodulating agents class showed the most adverse events reported in FAERS, which is consistent with the common understanding that anticancer drugs are more likely to cause various adverse events.

**Figure 3:**
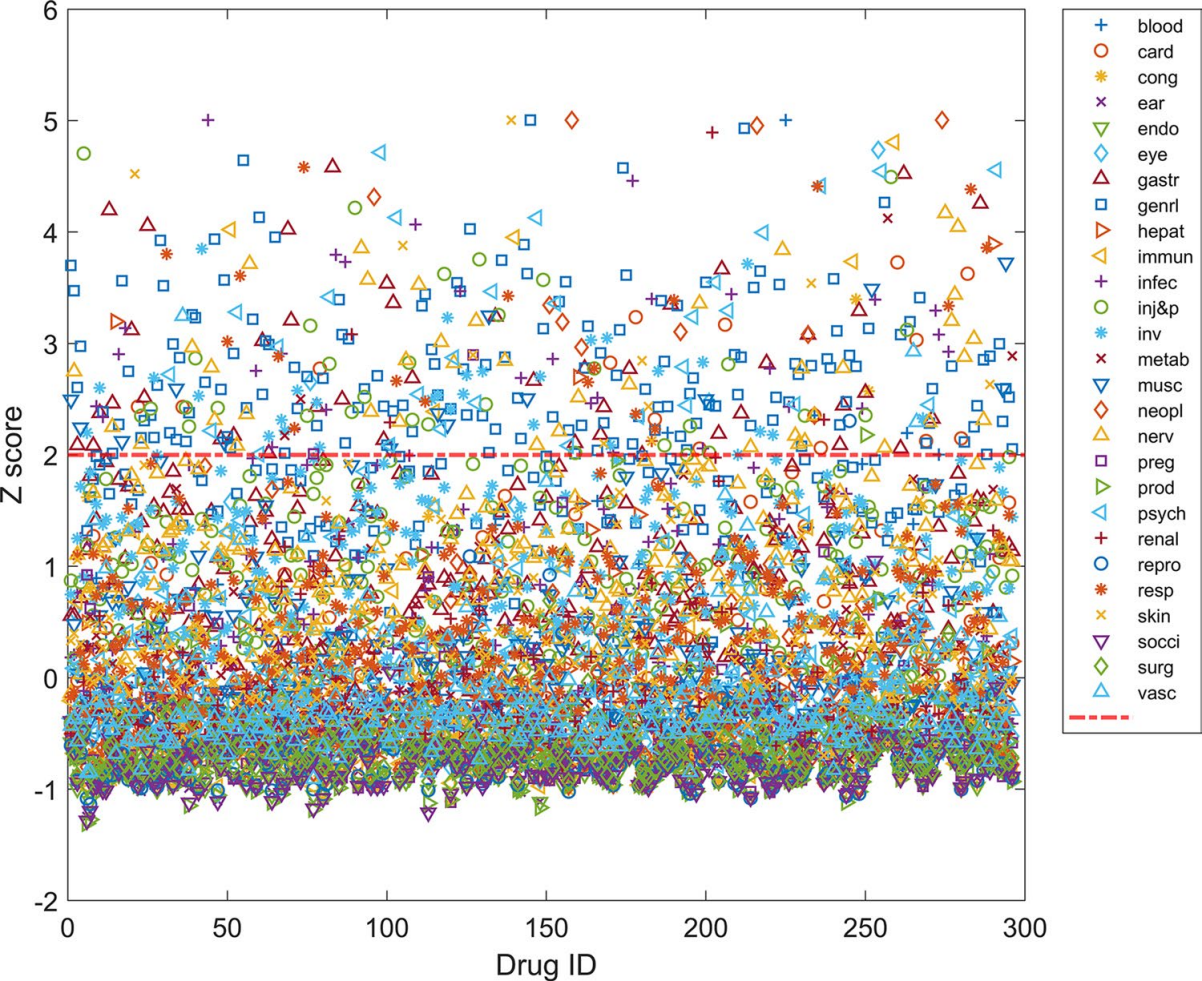
Z-scores of adverse events in SOCs. The x-axis indicates the drug ID which can be found in Supplementary Table 1. Colors and shapes for SOCs are given in the right legend in which the SOC abbreviations can be found in Supplementary Table 3. The red dashed line represents the threshold.

To quantitatively measure the relative frequency of adverse events as drug safety metrics, we calculated Z-scores of adverse events in SOCs for each drug. A higher Z-score for a drug indicates a greater likelihood of the occurrence of SOC adverse events. Z-scores on the 27 SOCs for the 296 drugs for COVID-19 treatment in clinical trials are shown in Supplementary Table 4 and are plotted in Fig. 3. Statistically, a Z-score of 2 indicates that a specific type of adverse event would likely occur 98% more often than all other adverse events associated with the drug. We defined an SOC as a significant type of adverse event for a drug if the Z-score was > 2 (above the red dash line in Fig. 3). The significant types of adverse events for the 296 drugs are listed in Supplementary Table 4 (red cells). To utilize Z-scores as a drug safety concern metric, we categorized Z-scores into 5 groups which are marked by colors in Supplementary Table 4: red for Z-scores > 2 indicating significantly frequent adverse events; light red for Z-scores between 1 and 2 showing frequent adverse events; gray for Z-scores between 0 and 1 marking slightly frequent adverse events; light green for Z-scores between − 1 and 0 denoting likely infrequent adverse events; green for Z-scores less than − 1 representing infrequent adverse events. As shown in Supplementary Table 4 and Fig. 3, most drugs show one or two significantly frequent SOCs of adverse events, indicating that caution may be needed when administering those drugs to COVID-19 patients who are vulnerable to such types of adverse events. However, lopinavir, enoxaparin, and fondaparinux did not show significantly frequent SOCs, suggesting that a wide group of patients could use these drugs with few to no adverse events. The Z-scores shown in Supplementary Table 4 could be used as a safety concern metric to inform the selection of optimal drugs for COVID-19 treatment based on clinical patient information. For example, hydroxychloroquine is a potential COVID-19 drug that is currently being extensively tested in clinical trials (119 trials registered in ClinicalTrials.gov). However, it has not been demonstrated to be effective for COVID-19 treatment^8,20^. Our study revealed that hydroxychloroquine very frequently causes adverse events pertaining to general disorders which belongs to the SOC of “general disorders and administration site conditions” (Z-score = 3.7) and “musculoskeletal and connective tissue disorders” (Z-score = 2.5), strongly suggesting that physicians need to consider these adverse events in benefit risk assessment before prescribing this drug to COVID-19 patients whose clinical information shows vulnerability to such adverse events. Rosenberg et al.^23^ conducted a retrospective multicenter cohort study of patients with COVID-19 treated with both hydroxychloroquine and azithromycin, hydroxychloroquine alone, azithromycin alone, or neither and found that cardiac arrest was more likely in patients who received both hydroxychloroquine and azithromycin, but not hydroxychloroquine alone and azithromycin alone, compared with patients who received neither drug, even after adjustment. Their findings are consistent with our results that cardiac disorder is a less frequently reported adverse event for hydroxychloroquine alone (Z-score =  − 0.4) and azithromycin alone (Z-score =  − 0.03). Thus, our Z-scores could be used to as a safety concern metric for drugs for treating COVID19 patients. Another example is chloroquine which is being tested in 12 clinical trials for COVID-19 treatment. Our safety analysis revealed that this drug frequently causes adverse events pertaining to cardiac disorders (Z-score = 2.4). Therefore, it is recommended that physicians avoid prescribing chloroquine to patients vulnerable to these types of adverse events, such as patients with hypertension.

Our safety concern metrics can be used for guidance in avoiding improper prescription of drugs to patients, and can also help to optimize treatment for COVID-19. For example, in patients with hypertension, our results (Supplementary Table 4) not only inform physicians to avoid prescription of trimetazidine (Z-score = 3.7), ulinastatin (Z-score = 3.6), adenosine (Z-score = 3.2), bivalirudin (Z-score = 3.2), tirofiban (Z-score = 3.0), dexmedetomidine (Z-score = 2.8), sevoflurane (Z-score = 2.8), chloroquine (Z-score = 2.4), angiotensin ii (Z-score = 2.4), molgramostim (Z-score = 2.4), amiodarone (Z-score = 2.3), propofol (Z-score = 2.1), verapamil (Z-score = 2.1), escin (Z-score = 2.1) and tenecteplase (Z-score = 2.1) because adverse events of cardiac disorders are significantly frequent with these drugs, but our results also suggest some other drugs like clavulanic acid (Z-score =  − 0.7) and tenofovir alafenamide (Z-score =  − 0.9) have less frequent adverse events of cardiac disorders compared to other reported adverse events of these drugs. This information can facilitate healthcare providers benefit-risk evaluations for these patients to make the best choice when selecting a drug among those that have a similar degree of efficacy.

Since pharmacokinetics and pharmacodynamics might differ between men and women, gender plays an important role in adverse events related to a drug. To help physicians understand potential adverse events specifically for female and male patients, we provided the Z-scores on 27 SOCs for female and male patients in Supplementary Tables 5 and 6, respectively. We identified 11,420,242 adverse events reported for 268 drugs for female patients and 6,033,116 adverse events for 269 drugs for male patients. The remaining adverse events were either reported without sex information or have sex values of “Intersex”, “Transgender”, “Prefer not to disclose” or “Unknown”. As shown in Supplementary Tables 5 and 6, the Z-scores of SOC adverse events for each identified drug were categorized into 5 groups with colors representing likelihood levels of the occurrence of SOC adverse events. These two tables could be used to inform selection of drugs for treating female and male COVID-19 patients. For example, for male patients, Supplementary Table 6 suggests lopinavir and ritonavir are relatively safe without any significant frequent adverse events reported. However, both drugs have significant frequent adverse events for female patients (Supplementary Table 5): injury, poisoning and procedural complications (Z-score = 3.2 and 2.1), informing that more caution is needed when prescribing these two drugs to female patients.

We examined whether the significantly frequent adverse events are distributed differently in the ATC classes of drugs. Significantly frequent adverse events for the 14 ATC classes of drugs are summarized in Supplementary Fig. 2. Both total and averaged significantly frequent adverse events were not dramatically different among the ATC classes of drugs except for the drugs in the ATC various class which contained fewer significantly adverse events than other classes of drugs. Although drugs in the ATC various class displayed less safety concern, our analysis did not support evaluating safety concerns by ATC classes. Our data suggests that the Z-score safety concern metrics for individual drugs in Supplementary Table 4 could be used to inform physicians when selecting optimal drugs for patients and identifying appropriate patients for a specific drug.

There are some limitations in this study. First, in our analysis, information regarding concomitant use of drugs and adverse events specifically resulting from drug-drug interactions were not captured. Second, our analysis did not dissect the sub-demographics (e.g., race) that could impact results; some drugs may show significant sex/race difference in adverse events. Third, we did not have access to the efficacy side of the equation for drug selection. If the health benefit of a drug outweighs its safety concern, drugs with severe toxicity and/or high Z-score may still be used in clinical practice. Fourth, dose was not included in our study because we had difficulties obtaining dose information from clinical trials. Furthermore, because the majority of the COVID-19 trials are still ongoing, our safety metrics did not include safety information from COVID-19 patients in these trials. Lastly, we did not consider the severity of the adverse events which is an important clinical consideration.

In summary, we investigated safety concerns for COVID-19 drugs by analyzing adverse events reported in FAERS. We found 406 drugs registered in ClinicalTrials.gov, of which 296 drugs showed 28,597,464 SOCs adverse events in FAERS. We calculated Z-scores for SOCs of adverse events to qualitatively measure safety concerns for 296 drugs. Physicians may need to be cautious when prescribing drugs with high Z-scores for adverse events in SOCs in which patients have vulnerabilities. New evidence is evolving every day on clinical outcomes and different treatment options for patients infected with SARS-CoV-2. Since our original analyses, additional options have become available for the management of COVID-19 (e.g. https://www.covid19treatmentguidelines.nih.gov/whats-new/). Physicians should be cognizant of the Z-scores as the treatment algorithms evolve.

## Methods

### Identification of drugs for COVID-19 treatment in clinical practice

ClincialTrials.gov is a registry database for clinical studies which provides researchers access to ongoing COVID-19 clinical trials worldwide. On June 3, 2021, we downloaded 5849 COVID-19-related clinical trials in a CSV file from ClinicalTrials.gov. The csv file contained information such as clinical trial title, recruitment status, study results and details of interventions. To identify the potential drugs in the downloaded clinical trials, we performed the following steps. First, we excluded trials that were marked as withdrawn, suspended, and terminated in the recruitment status to focus on promising COVID-19 drugs. Then, for the remaining retrieved clinical trials we only included clinical trials that listed drugs as interventions. Since drug names in clinical trials are listed along with drug dosage and form in the Intervention field and they are not standardized, they may be full names, trade names, abbreviations, active ingredients or synonyms. We compiled a comprehensive list of drug names using synonyms and common drug names from DrugBank^19^. This comprehensive list of drug names was then used to identify drugs used in clinical trials. After these steps, we identified 406 approved or investigational drugs from 1075 clinical trials.

To further examine drugs at the organ level, we grouped the drugs using anatomical therapeutic chemical (ATC) codes. The ATC classification system divides drugs into different groups according to the organ or system on which they act. There are five levels in the ATC classification system. The first level has 14 codes corresponding to 14 anatomical main groups. ATC codes for drugs were extracted from the National Center for Biomedical Ontology (NCBO) BioPortal ontologies^24^. In our study, 308 ATC codes were found for 237 drugs and the drugs were classified into 14 anatomical groups using the first letter of their ATC codes. The reason that a drug may have more than one ATC code is that a bottom-level code only stands for a single use and a drug may have multiple uses. Some drugs investigated that didn’t have ATC codes assigned were not considered in our ATC analysis.

### Extraction of adverse events from the FAERS database

FAERS is a self-reporting adverse events system provided by the FDA. It is widely used to study relationships between drugs and adverse events due to the large number of adverse event reports in the database. To investigate the potential link between COVID-19 drugs and adverse events, we downloaded the publicly available FAERS database from January 2004 to March 2021. Since FAERS is a self-reporting system, it contains duplicate and incomplete reports. To improve the data quality, we pre-processed duplicate and incomplete reports in our analysis. Only the latest version of the report was retained for duplicate reports with the same case ID, drug name, and adverse events. The incomplete reports were also removed if we could not locate the report case ID, drug name and its adverse events. To reduce the risk of false positives, only “primary suspected” and “secondary suspected” drugs for the reported adverse event were considered in our analysis. After cleaning up the database, we used the drugs names extracted from clinical trials to find their adverse events. Since the drug names in FAERS are not standardized, and may be full names, trade names or abbreviations, we used the active ingredients, commonly used drug name, and synonyms of the extracted drugs to do a thorough search. The synonym and commonly used drug names were taken from DrugBank. Finally, we identified 28,597,464 adverse events reported for the drugs that were used in the clinical trials registered on ClinicalTrials.gov.

The adverse events in the FAERS database are reported using preferred terms (PTs) or lowest level terms (LLTs) in MedDRA^25,26^. We used MedDRA V22.1 to group the reported LLTs and PTs into 27 SOCs. The adverse events that were not identified as PTs or LLTs in MedDRA V22.1 were discarded in our analysis. SOCs are the highest level of MedDRA hierarchy. LLT and PTs are grouped into SOCs by their physiological system, manifestation site or purpose. Since one PT may be mapped to different SOCs, only the primary SOC was considered in the analysis. The 27 SOCs and their abbreviations used in this study are listed in Supplementary Table 3. The number of adverse events of each SOC for each drug was then aggregated from the drug-event combination.

### Statistical analysis

To examine whether the occurrence of an SOC of adverse events was significantly frequent for a drug, we calculated the Z-score for the SOC and the drug using the following equation.

$$ Z_{i}^{j}  = ~\frac{{N_{i}^{j}  - \upmu _{j} }}{{\upsigma _{j} }} $$

where ${\text{N}}_{i}^{j}$ is number of adverse events in SOC *i* that were found in FAERS for drug *j*, $\upmu _{j}$ is average number of adverse events in a SOC for drug *j* and is calculated by dividing the total number of adverse events by 27 (number of SOCs), and $\upsigma _{j}$ is the standard deviation of adverse events for drug *j.* If the Z-score for a SOC for a specific drug is larger than 2, this SOC is a significantly frequent adverse event among all 27 SOCs. Similarly, this SOC is a frequent adverse event if its Z-score is between 1 and 2, and a slightly frequent adverse event if Z-score is between 0 and 1; likely infrequent adverse event if Z-score is between -1 and 0 and an infrequent adverse event if Z-score is less than − 1.

All data processing and statistical analyses were performed using in-house Python scripts in Python 3.6 (Python Software Foundation, http://python.org) (Python Software Foundation, Beaverton, OR, USA).

## Supplementary Information

Supplementary Information 1.

Supplementary Information 2.

Supplementary Information 3.

Supplementary Information 4.
