# Supplementary material for: Informing selection of drugs for COVID-19 treatment through adverse events analysis
Source: Sci Rep. 2021 Jul 7;11:14022. doi: 10.1038/s41598-021-93500-5 (PMC8263777; doi:10.1038/s41598-021-93500-5)
Supplement: Supplementary file 1 — Supplementary Information 1. [file 41598_2021_93500_MOESM1_ESM.docx]

Informing selection of drugs for COVID-19 treatment through adverse events analysis

Wenjing Guo^1^, Bohu Pan^1^, Sugunadevi Sakkiah^1^, Zuowei Ji^1^, Gokhan Yavas^1^, Yanhui Lu^2^, Takashi E. Komatsu^2^, Madhu Lal-Nag^2^, Weida Tong^1^, Tucker A. Patterson^1^, Huixiao Hong^1*^

^1^ National Center for Toxicological Research, U.S. Food & Drug Administration, 3900 NCTR Road, Jefferson, AR 72079

^2^ Center for Drug Evaluation and Research, U.S. Food & Drug Administration, 10903 New Hampshire Avenue, Silver Spring, MD 20993

^*^ Correspondence: [huixiao.hong@fda.hhs.gov](mailto:huixiao.hong@fda.hhs.gov)

***Disclaimer:*** *The views presented in this article do not necessarily reflect those of the US Food and Drug Administration.*

Supplementary Figure 1 ……………………………………………………………………page 2

Supplementary Figure 2 ……………………………………………………………………page 3

Supplementary Table 1 …………………………………………………………………….page 4

Supplementary Table 2 …………………………………………………………………….page 21

Supplementary Table 3 …………………………………………………………………….page 23

**B**

**A**


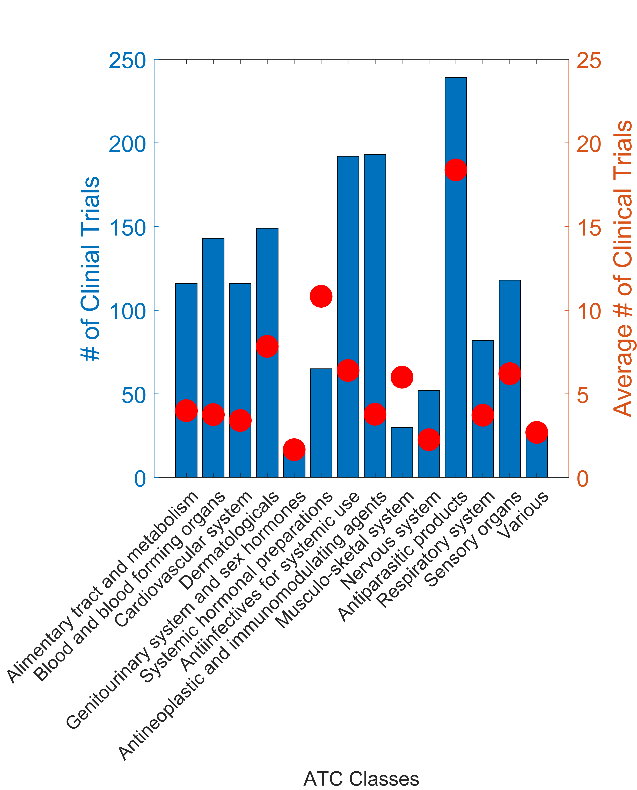

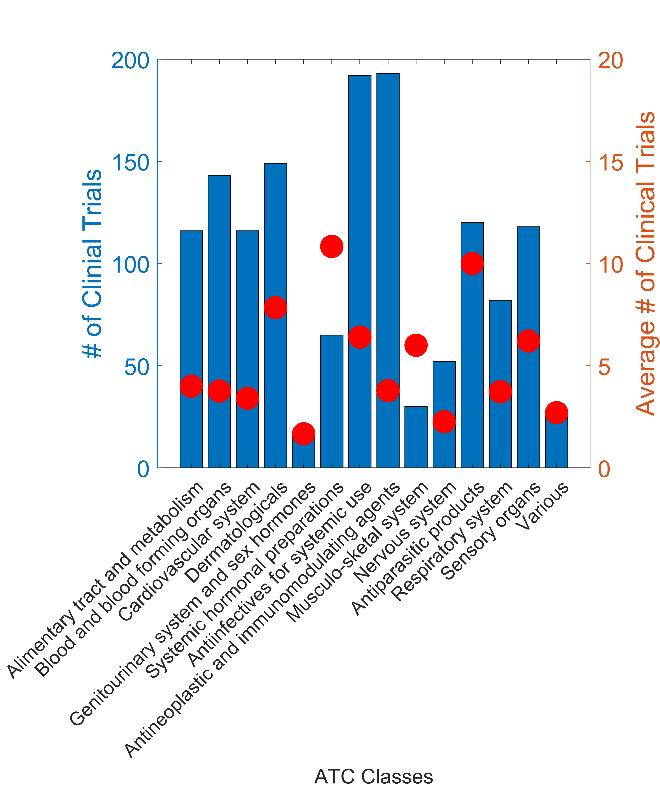


**Supplementary Figure 1. Summary of clinical trials for ATC classes of drugs.** Number of clinical trials (left y-axis) and average number of clinical trials (right y-axis) for 14 Anatomical Therapeutic Chemical (ATC) classes. The red solid circles represent average number of clinical trials. Hydroxychloroquine in antiparasitic products class has 119 clinical trials, which is way larger than that of other drugs. To see the impact of hydroxychloroquine on the number of clinical trial studies, number of clinical trials was plotted with hydroxychloroquine in the antiparasitic products group (**A**) and without hydroxychloroquine in the antiparasitic products group(**B**). Average number of clinical trials are calculated by dividing number of clinical trials by number of drugs in each ATC classes.


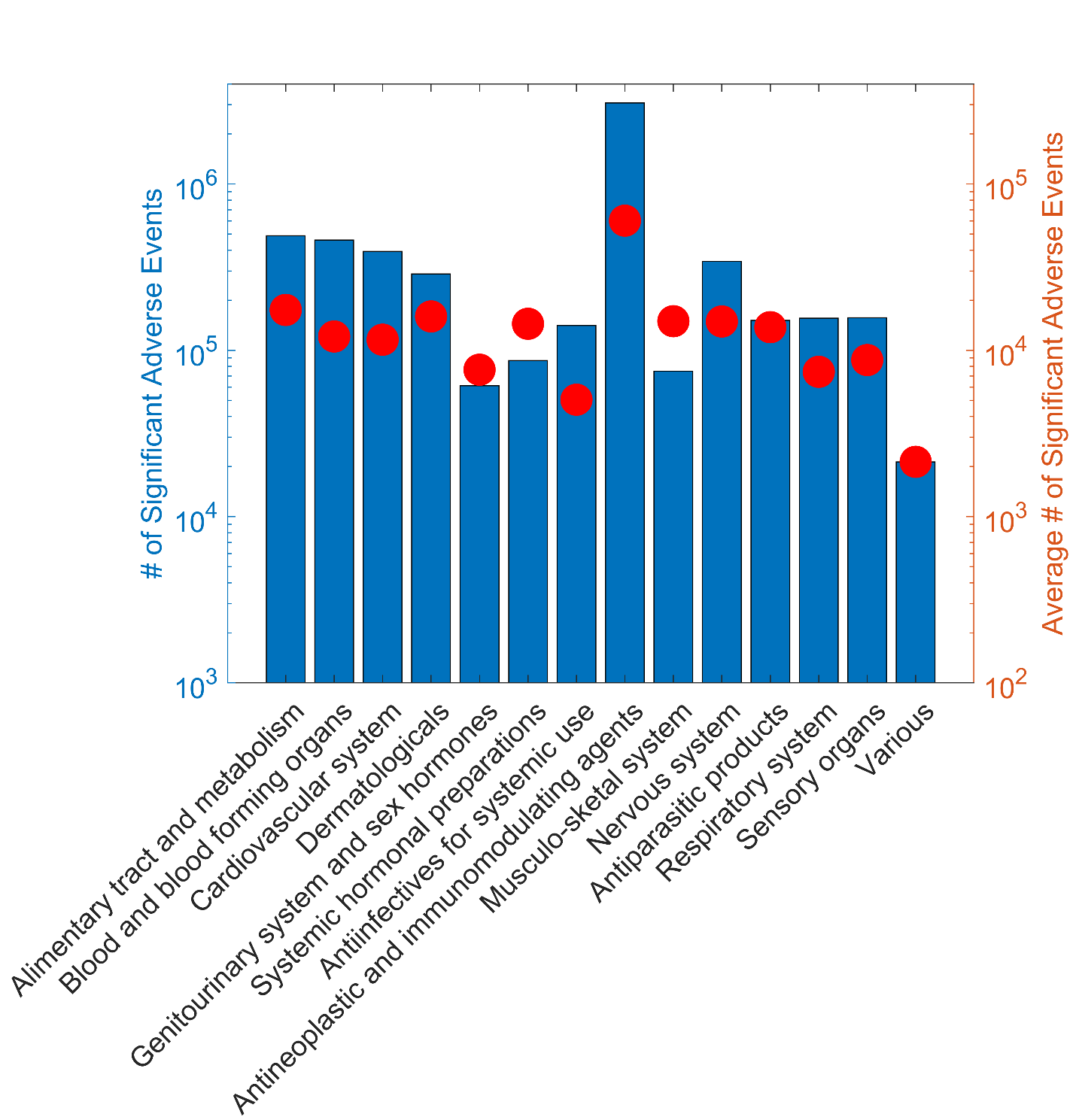


**Supplementary Figure 2. Summary of significantly frequent adverse events in ATC classes**. Number of significantly frequent adverse events (left y-axis) and average number of significantly frequent adverse events (right y-axis) are plotted for the 14 Anatomical Therapeutic Chemical (ATC) classes (x-axis). The red solid circles represent average number of significantly frequent adverse events.

**Supplementary Table 1**. Drugs used for COVID-19 treatment in ClinicalTrials.gov

| Drug ID | Drug Name | # of Clinical Trials | Clinical Trial ID in ClinicalTrials.gov |
| --- | --- | --- | --- |
| Drug 1 | hydroxychloroquine | 119 | NCT04460547,NCT04715295,NCT04343768,NCT04359615,NCT04359316,NCT04329832,NCT04359953,NCT04397328,NCT04350684,NCT04350671,NCT04466540,NCT04328285,NCT04352933,NCT04394442,NCT04443725,NCT04858633,NCT04351620,NCT04334382,NCT04391127,NCT04333225,NCT04351724,NCT04332094,NCT04344444,NCT04429867,NCT04331834,NCT04466280,NCT04370782,NCT04315896,NCT04354870,NCT04668469,NCT04712279,NCT04354428,NCT04372017,NCT04344457,NCT04573153,NCT04411433,NCT04336332,NCT04349592,NCT04347980,NCT04437693,NCT04395768,NCT04387760,NCT04860284,NCT04355026,NCT04446104,NCT04353336,NCT04779047,NCT04328272,NCT04714515,NCT04318444,NCT04318015,NCT04438837,NCT04390061,NCT04345653,NCT04477083,NCT04405921,NCT04377646,NCT04554979,NCT04261517,NCT04458948,NCT04330144,NCT04746365,NCT04359537,NCT04349410,NCT04410562,NCT04328961,NCT04590274,NCT04341207,NCT04368351,NCT04334512,NCT04420247,NCT04435587,NCT04338698,NCT04303507,NCT04352946,NCT04386070,NCT04334148,NCT04363450,NCT04414241,NCT04370015,NCT04434144,NCT04332991,NCT04366089,NCT04350281,NCT04363827,NCT04384380,NCT04403100,NCT04376814,NCT04355052,NCT04334928,NCT04605588,NCT04381936,NCT04315948,NCT04335084,NCT04652648,NCT04321616,NCT04342169,NCT04380818,NCT04340349,NCT02735707,NCT04394182,NCT04597775,NCT04400019,NCT04328467,NCT04384458,NCT04497519,NCT04322123,NCT04321278,NCT04322396,NCT04336748,NCT04383717,NCT04389320,NCT04461353,NCT04347031,NCT04390152,NCT04308668,NCT04788355,NCT04316377, NCT04330495 |
| Drug 2 | ivermectin | 63 | NCT04460547,NCT04646109,NCT04681053,NCT04381884,NCT04391127,NCT04405843,NCT04429711,NCT04407130,NCT04668469,NCT04712279,NCT04510233,NCT04894721,NCT04729140,NCT04591600,NCT04739410,NCT04885530,NCT04523831,NCT04529525,NCT04673214,NCT04446104,NCT04343092,NCT04779047,NCT04714515,NCT04886362,NCT04438850,NCT04727424,NCT04602507,NCT04746365,NCT04425707,NCT04703205,NCT04510194,NCT04784481,NCT04551755,NCT04834115,NCT04392713,NCT04445311,NCT04392427,NCT04422561,NCT04403555,NCT04374019,NCT04351347,NCT04399746,NCT04407507,NCT04390022,NCT04832945,NCT04716569,NCT04701710,NCT04472585,NCT04527211,NCT04703608,NCT04891250,NCT04632706,NCT04435587,NCT04635943,NCT04447235,NCT04373824,NCT04434144,NCT04836299,NCT04747678,NCT04482686,NCT04425863,NCT04384458,NCT04431466 |
| Drug 3 | azithromycin | 48 | NCT04715295,NCT04359316,NCT04329832,NCT04359953,NCT04334382,NCT04332094,NCT04344444,NCT04370782,NCT04381962,NCT04622891,NCT04332107,NCT04354428,NCT04363060,NCT04411433,NCT04336332,NCT04349592,NCT04395768,NCT04673214,NCT04328272,NCT04339426,NCT04405921,NCT04458948,NCT04349410,NCT04590274,NCT04399746,NCT04341207,NCT04369365,NCT04457609,NCT04729491,NCT04368351,NCT04334512,NCT04699097,NCT04338698,NCT04371107,NCT04434144,NCT04366089,NCT04355052,NCT04381936,NCT04380818,NCT04394182,NCT04441424,NCT04322123,NCT04321278,NCT04322396,NCT04383717,NCT04347031,NCT04390152,NCT03871491 |
| Drug 4 | tocilizumab | 47 | NCT04445272,NCT04345445,NCT04479358,NCT04317092,NCT04330638,NCT04412772,NCT04331795,NCT04332094,NCT04377659,NCT04320615,NCT04372186,NCT04409262,NCT04600141,NCT04730323,NCT04678739,NCT04359667,NCT04310228,NCT04377750,NCT04412291,NCT04335305,NCT04779047,NCT04435717,NCT04693026,NCT04377503,NCT04424056,NCT04349410,NCT04363853,NCT04356937,NCT04361032,NCT04734678,NCT04690920,NCT04873141,NCT04893031,NCT04339712,NCT04492501,NCT04560205,NCT04381936,NCT04306705,NCT04380818,NCT02735707,NCT04394182,NCT04315480,NCT04871854,NCT04519385,NCT04476979,NCT04347031,NCT04331808 |
| Drug 5 | favipiravir | 33 | NCT04336904,NCT04434248,NCT04464408,NCT04359615,NCT04542694,NCT04532931,NCT04475991,NCT04501783,NCT04358549,NCT04474457,NCT04448119,NCT04425460,NCT04828564,NCT04349241,NCT04346628,NCT04411433,NCT04558463,NCT04310228,NCT04600895,NCT04818320,NCT04387760,NCT04694612,NCT04402203,NCT04333589,NCT04499677,NCT04351295,NCT04445467,NCT04600999,NCT04376814,NCT04373733,NCT04319900,NCT04727775,NCT04407000 |
| Drug 6 | ritonavir | 33 | NCT04364022,NCT04343768,NCT04350684,NCT04350671,NCT04328285,NCT04372628,NCT04351724,NCT04331470,NCT04459286,NCT04354428,NCT04286503,NCT04779047,NCT04738045,NCT04466241,NCT04499677,NCT04521400,NCT04295551,NCT04425382,NCT04403100,NCT04376814,NCT04315948,NCT04255017,NCT04261907,NCT04380818,NCT02735707,NCT04394182,NCT04321174,NCT04276688,NCT04390152,NCT04275388,NCT04252885,NCT04909853,NCT04386876 |
| Drug 7 | lopinavir | 31 | NCT04364022,NCT04343768,NCT04350684,NCT04350671,NCT04328285,NCT04372628,NCT04351724,NCT04331470,NCT04354428,NCT04286503,NCT04779047,NCT04738045,NCT04466241,NCT04499677,NCT04521400,NCT04295551,NCT04425382,NCT04403100,NCT04376814,NCT04315948,NCT04255017,NCT04261907,NCT04380818,NCT02735707,NCT04394182,NCT04321174,NCT04276688,NCT04390152,NCT04275388,NCT04252885,NCT04386876 |
| Drug 8 | heparin | 30 | NCT04406389,NCT04466670,NCT04635241,NCT04486508,NCT04545541,NCT04344756,NCT04487990,NCT04409834,NCT04528888,NCT04394377, NCT04485429,NCT04600141,NCT04393805,NCT04359212,NCT04723563,NCT04542408,NCT04655586,NCT04505774,NCT04372589,NCT04743011,NCT04530578,NCT04367831,NCT04787510,NCT04842292,NCT04397510,NCT04584580,NCT04511923,NCT04380818,NCT04394182,NCT04490239 |
| Drug 9 | dexamethasone | 27 | NCT04640168,NCT04707534,NCT04784559,NCT04513184,NCT04347980,NCT04344730,NCT04832880,NCT04726098,NCT04663555,NCT04509973,NCT04909918,NCT04499313,NCT04603729,NCT04561180,NCT04636671,NCT04890626,NCT04836780,NCT04452565,NCT04834375,NCT04445506,NCT04780581,NCT04425863,NCT04826822,NCT04519385,NCT04765371,NCT04476979,NCT04545242 |
| Drug 10 | colchicine | 25 | NCT04359095,NCT04392141,NCT04375202,NCT04667780,NCT04355143,NCT04527562,NCT04360980,NCT04654416,NCT04756128,NCT04724629,NCT04350320,NCT04492358,NCT04818489,NCT04328480,NCT04516941,NCT04416334,NCT04381936,NCT04867226,NCT04322565,NCT04403243,NCT04324463,NCT04539873,NCT04363437,NCT04367168,NCT04322682 |
| Drug 11 | methylprednisolone | 25 | NCT04345445,NCT04485429,NCT04244591,NCT04329650,NCT04673162,NCT04273321,NCT04438980,NCT04355247,NCT04341038,NCT04909918,NCT04499313,NCT04603729,NCT04377503,NCT04349410,NCT04636671,NCT04826588,NCT04374071,NCT04528888,NCT04847687,NCT04559113,NCT04263402,NCT04323592,NCT04780581,NCT04343729,NCT03852537 |
| Drug 12 | enoxaparin | 25 | NCT04406389,NCT04466670,NCT04492254,NCT04427098,NCT04366960,NCT04483960,NCT04512079,NCT04640181,NCT04486508,NCT04377997,NCT04530578,NCT04367831,NCT04646655,NCT04345848,NCT04736901,NCT04354155,NCT04401293,NCT04409834,NCT04400799,NCT04408235,NCT04508439,NCT04528888,NCT04394377,NCT04373707,NCT04425863 |
| Drug 13 | nitazoxanide | 22 | NCT04532931,NCT04486313,NCT04348409,NCT04463264,NCT04343248,NCT04523090,NCT04359680,NCT04459286,NCT04746183,NCT04435314,NCT04441398,NCT04423861,NCT04561063,NCT04552483,NCT04392427,NCT04498936,NCT04729491,NCT04561219,NCT04382846,NCT04788407,NCT04605588,NCT04406246 |
| Drug 14 | ascorbic acid | 22 | NCT04357782,NCT04344184, NCT04363216,NCT04354428,NCT04710329,NCT04328961, NCT04664010,NCT04712357,NCT04279197,NCT04395768,NCT04446104,NCT04370288,NCT04468139,NCT04780061,NCT04401150,NCT04334512,NCT04570254,NCT04335084,NCT04482686,NCT02735707,NCT04682574,NCT03680274 |
| Drug 15 | camostat | 19 | NCT04353284,NCT04583592,NCT04455815,NCT04662086,NCT04662073,NCT04524663,NCT04625114,NCT04608266,NCT04518410,NCT04730206,NCT04374019,NCT04652765,NCT04435015,NCT04530617,NCT04681430,NCT04750759,NCT04321096,NCT04355052,NCT04470544 |
| Drug 16 | baricitinib | 16 | NCT04321993,NCT04401579,NCT04640168,NCT04362943,NCT04421027,NCT04346147,NCT04832880,NCT04693026,NCT04358614,NCT04390464,NCT04393051,NCT04890626,NCT04381936,NCT04320277,NCT04399798,NCT04891133 |
| Drug 17 | anakinra | 15 | NCT04362943,NCT04330638,NCT04443881,NCT04680949,NCT04412291,NCT04362111,NCT04424056,NCT04603742,NCT04357366,NCT04339712,NCT04643678,NCT04381936,NCT02735707,NCT04341584,NCT04148430 |
| Drug 18 | ruxolitinib | 14 | NCT04355793,NCT04414098,NCT04337359,NCT04334044,NCT04331665,NCT04362137,NCT04361903,NCT04338958,NCT04424056,NCT04374149,NCT04581954,NCT04359290,NCT04348695,NCT04403243 |
| Drug 19 | cholecalciferol | 13 | NCT04552951,NCT04636086,NCT04535791,NCT04399746,NCT04502667,NCT04733625,NCT04344041,NCT03188796, NCT04395768,NCT04780061,NCT04482673,NCT04482686,NCT04489628 |
| Drug 20 | acetylsalicylic acid | 13 | NCT04466670,NCT04808895,NCT04368377, NCT04333407,NCT04498273,NCT04483960,NCT04410328,NCT04365309,NCT04363840,NCT04381936,NCT02735707,NCT04324463,NCT04425863 |
| Drug 21 | sulfate ion | 13 | NCT04681079,NCT04354428,NCT04336332,NCT04446104,NCT04345653,NCT04359537,NCT04328961,NCT04384380,NCT04403100,NCT04380818,NCT04340349,NCT04461353,NCT04316377 |
| Drug 22 | chloroquine | 12 | NCT04443270,NCT04351724,NCT04331600,NCT04328493,NCT04627467,NCT04286503,NCT04353336,NCT04447534,NCT04420247,NCT04303507,NCT04342650,NCT04323527 |
| Drug 23 | zinc | 11 | NCT04621149,NCT04395768,NCT04446104,NCT04468139,NCT04780061,NCT04377646,NCT04447534,NCT04472585,NCT04334512,NCT04335084,NCT04482686 |
| Drug 24 | doxycycline | 11 | NCT04715295,NCT04370782,NCT04407130,NCT04729140,NCT04523831,NCT04371952,NCT04551755,NCT04349410,NCT04434144,NCT04482686, NCT04584567 |
| Drug 25 | rivaroxaban | 11 | NCT04333407,NCT04757857,NCT04715295,NCT04416048,NCT04351724,NCT04508023,NCT04640181,NCT04662684,NCT04736901,NCT04394377,NCT04324463 |
| Drug 26 | nitric oxide | 10 | NCT04388683,NCT04383002,NCT04601077,NCT04460183,NCT04338828,NCT04305457,NCT04312243,NCT04842331,NCT04306393,NCT03331445 |
| Drug 27 | famotidine | 9 | NCT04370262,NCT04389567,NCT04621149,NCT04488081,NCT04504240,NCT04724720,NCT04836806,NCT04565392,NCT04545008 |
| Drug 28 | losartan | 9 | NCT04335123,NCT04643691,NCT04328012,NCT04606563,NCT04349410,NCT04447235,NCT04312009,NCT04311177,NCT04340557 |
| Drug 29 | sarilumab | 9 | NCT04327388, NCT04315298,NCT04661527,NCT04357808,NCT04386239,NCT04357860,NCT02735707,NCT04327388,NCT04324073 |
| Drug 30 | sofosbuvir | 9 | NCT04532931,NCT04535869,NCT04443725,NCT04530422,NCT04561063,NCT04497649,NCT04498936,NCT04460443,NCT04773756 |
| Drug 31 | oxygen | 9 | NCT04842448,NCT04344730,NCT04338828,NCT04500626,NCT04371601,NCT04327505,NCT04380818,NCT04394182,NCT04425031 |
| Drug 32 | oseltamivir | 8 | NCT04558463,NCT04516915,NCT04371601,NCT04457609,NCT04338698,NCT04255017,NCT04261270,NCT02735707 |
| Drug 33 | daclatasvir | 8 | NCT04532931,NCT04468087,NCT04535869,NCT04443725,NCT04561063,NCT04497649,NCT04460443,NCT04773756 |
| Drug 34 | atorvastatin | 8 | NCT04333407,NCT04380402,NCT04801940,NCT04631536,NCT04466241,NCT04486508,NCT04900155,NCT04904536 |
| Drug 35 | vitamin d | 8 | NCT04372017,NCT04709744,NCT04411446,NCT04334512,NCT04363840,NCT04525820,NCT04335084,NCT04621058 |
| Drug 36 | angiotensin ii | 7 | NCT04357535,NCT04591210,NCT04408326, NCT04394117,NCT04353596,NCT04591210,NCT04364984 |
| Drug 37 | apixaban | 7 | NCT04498273,NCT04512079,NCT04650087,NCT04801940,NCT04746339,NCT04736901,NCT04788355 |
| Drug 38 | emtricitabine | 7 | NCT04359095,NCT04712357,NCT04405271,NCT04519125,NCT04685512,NCT04890626,NCT04334928 |
| Drug 39 | interferon beta-1a | 7 | NCT04460547,NCT04343768,NCT04492475,NCT04350671,NCT04330690,NCT04647669,NCT04315948 |
| Drug 40 | water | 7 | NCT04410159,NCT04707742,NCT04469491,NCT04842721,NCT04409873,NCT04721457,NCT04872686 |
| Drug 41 | telmisartan | 7 | NCT04359953,NCT04355936,NCT04360551,NCT04466241,NCT04510662,NCT04356495,NCT04715763 |
| Drug 42 | nafamostat | 7 | NCT04628143,NCT04390594,NCT04473053,NCT04623021,NCT04483960,NCT04418128,NCT04352400 |
| Drug 43 | tretinoin | 6 | NCT04396067, NCT04353180,NCT04623385,NCT04396067,NCT04730895,NCT04578236 |
| Drug 44 | clazakizumab | 6 | NCT04381052,NCT04494724,NCT04343989,NCT04363502,NCT04348500,NCT04659772 |
| Drug 45 | melatonin | 6 | NCT04474483,NCT04784754,NCT04409522,NCT04568863,NCT04353128,NCT04570254 |
| Drug 46 | interferon beta-1b | 6 | NCT04465695,NCT04343768,NCT04647695,NCT04494399,NCT04350281,NCT04276688 |
| Drug 47 | tinzaparin | 6 | NCT04808882,NCT04483960,NCT04344756,NCT04593654,NCT04730856,NCT04412304 |
| Drug 48 | ribavirin | 6 | NCT04664010,NCT04828564,NCT04494399,NCT04392427,NCT04605588,NCT04276688 |
| Drug 49 | tofacitinib | 5 | NCT04750317,NCT04415151,NCT04469114,NCT04390061,NCT04332042 |
| Drug 50 | quercetin | 5 | NCT04578158,NCT04861298,NCT04468139,NCT04851821,NCT04853199 |
| Drug 51 | chloride ion | 5 | NCT04445285,NCT04723563,NCT04584684,NCT04723446,NCT04721457 |
| Drug 52 | sargramostim | 5 | NCT04326920,NCT04411680,NCT04642950,NCT04707664,NCT04400929 |
| Drug 53 | fluvoxamine | 5 | NCT04718480,NCT04342663,NCT04727424,NCT04510194,NCT04668950 |
| Drug 54 | bromhexine | 5 | NCT04355026,NCT04273763,NCT04405999,NCT04340349,NCT04424134 |
| Drug 55 | hydrogen peroxide | 5 | NCT04603794,NCT04584684,NCT04723446,NCT04721457,NCT04659928 |
| Drug 56 | sodium chloride | 5 | NCT04647604,NCT04364763,NCT04847141,NCT04784767,NCT04348305 |
| Drug 57 | cannabidiol | 5 | NCT04731116,NCT04615949,NCT04504877,NCT04467918, NCT04686539 |
| Drug 58 | canakinumab | 5 | NCT04348448,NCT04476706,NCT04362813,NCT04365153,NCT04510493 |
| Drug 59 | prednisone | 5 | NCT04451174,NCT04534478,NCT04795583,NCT04551781,NCT04492358 |
| Drug 60 | naltrexone | 5 | NCT04604678,NCT04756128,NCT04604704,NCT04708327,NCT04365985 |
| Drug 61 | pirfenidone | 4 | NCT04653831,NCT04607928,NCT04856111,NCT04282902 |
| Drug 62 | infliximab | 4 | NCT04425538,NCT04593940,NCT04734678,NCT04381936 |
| Drug 63 | imatinib | 4 | NCT04394416,NCT04346147,NCT04422678,NCT04794088 |
| Drug 64 | maraviroc | 4 | NCT04441385,NCT04475991,NCT04435522,NCT04710199 |
| Drug 65 | acalabrutinib | 4 | NCT04380688,NCT04346199,NCT04647669,NCT04564040 |
| Drug 66 | montelukast | 4 | NCT04389411,NCT04718285,NCT04714515,NCT04695704 |
| Drug 67 | cyclosporine | 4 | NCT04540926, NCT04412785,NCT04392531,NCT04492891 |
| Drug 68 | spironolactone | 4 | NCT04643691,NCT04424134,NCT04826822,NCT04345887 |
| Drug 69 | nintedanib | 4 | NCT04541680,NCT04856111,NCT04338802,NCT04619680 |
| Drug 70 | clopidogrel | 4 | NCT04333407,NCT04409834,NCT02735707,NCT04368377 |
| Drug 71 | dornase alfa | 4 | NCT04387786,NCT04355364,NCT04359654,NCT04402970 |
| Drug 72 | phosphate ion | 4 | NCT04443270,NCT04331600,NCT04328493,NCT04286503 |
| Drug 73 | metformin | 4 | NCT04604678,NCT04727424,NCT04510194,NCT04625985 |
| Drug 74 | ciclesonide | 4 | NCT04330586,NCT04377711,NCT04381364,NCT04356495 |
| Drug 75 | defibrotide | 4 | NCT04335201,NCT04652115,NCT04348383,NCT04530604 |
| Drug 76 | sodium bicarbonate | 4 | NCT04374591,NCT04655716,NCT04806061,NCT04530448 |
| Drug 77 | atazanavir | 3 | NCT04468087,NCT04459286,NCT04452565 |
| Drug 78 | fostamatinib | 3 | NCT04579393,NCT04581954,NCT04629703 |
| Drug 79 | sevoflurane | 3 | NCT04415060,NCT04359862,NCT04355962 |
| Drug 80 | nivolumab | 3 | NCT04413838,NCT04343144,NCT04356508 |
| Drug 81 | acetylcysteine | 3 | NCT04374461,NCT04792021,NCT04419025 |
| Drug 82 | ethanol | 3 | NCT04723446, NCT04554433,NCT04584684 |
| Drug 83 | isotretinoin | 3 | NCT04361422,NCT04353180,NCT04389580 |
| Drug 84 | prednisolone | 3 | NCT04657484,NCT04765371,NCT03708718 |
| Drug 85 | tenofovir disoproxil | 3 | NCT04685512,NCT04890626,NCT04334928 |
| Drug 86 | bevacizumab | 3 | NCT04305106,NCT04344782,NCT04275414 |
| Drug 87 | bcg vaccine | 3 | NCT04328441,NCT04534803,NCT04327206 |
| Drug 88 | chlorhexidine | 3 | NCT04603794,NCT04584684,NCT04723446 |
| Drug 89 | tenofovir | 3 | NCT04359095,NCT04712357,NCT04519125 |
| Drug 90 | levamisole | 3 | NCT04331470,NCT04360122,NCT04383717 |
| Drug 91 | ibrutinib | 3 | NCT04375397,NCT04665115,NCT04439006 |
| Drug 92 | methylene blue | 3 | NCT04376788,NCT04619290,NCT04635605 |
| Drug 93 | nicotine | 3 | NCT04583410,NCT04598594,NCT04608201 |
| Drug 94 | dipyridamole | 3 | NCT04424901,NCT04410328,NCT04391179 |
| Drug 95 | dextrose, unspecified form | 3 | NCT04664010,NCT04328272,NCT04395456 |
| Drug 96 | pioglitazone | 3 | NCT04604223,NCT04535700,NCT04473274 |
| Drug 97 | hydrocortisone | 3 | NCT04456439,NCT04348305,NCT02735707 |
| Drug 98 | cysteine | 3 | NCT04455243,NCT04545008,NCT04570254 |
| Drug 99 | dalteparin | 3 | NCT04483960,NCT04593654,NCT04412304 |
| Drug 100 | apremilast | 2 | NCT04590586,NCT02735707 |
| Drug 101 | cobicistat | 2 | NCT04425382,NCT04252274 |
| Drug 102 | honey | 2 | NCT04767087,NCT04347382 |
| Drug 103 | hesperidin | 2 | NCT04715932,NCT04452799 |
| Drug 104 | pentoxifylline | 2 | NCT04433988,NCT04570254 |
| Drug 105 | microcrystalline cellulose | 2 | NCT04780061,NCT04435015 |
| Drug 106 | lidocaine | 2 | NCT04609865,NCT04885777 |
| Drug 107 | sirolimus | 2 | NCT04341675,NCT04461340 |
| Drug 108 | duvelisib | 2 | NCT04372602,NCT04487886 |
| Drug 109 | dapagliflozin | 2 | NCT04350593,NCT04393246 |
| Drug 110 | amantadine | 2 | NCT04854759,NCT04894617 |
| Drug 111 | artesunate | 2 | NCT04387240,NCT04374019 |
| Drug 112 | ambrisentan | 2 | NCT04393246,NCT04771000 |
| Drug 113 | darunavir | 2 | NCT04425382,NCT04252274 |
| Drug 114 | deferoxamine | 2 | NCT04333550,NCT04361032 |
| Drug 115 | coconut oil | 2 | NCT04849637,NCT04594330 |
| Drug 116 | crizanlizumab | 2 | NCT04435184,NCT03474965 |
| Drug 117 | cyproheptadine | 2 | NCT04876573,NCT04820751 |
| Drug 118 | antithrombin iii human | 2 | NCT04651400,NCT04899232 |
| Drug 119 | artemisinin | 2 | NCT04801017,NCT04387240 |
| Drug 120 | bicalutamide | 2 | NCT04509999,NCT04652765 |
| Drug 121 | chlorpromazine | 2 | NCT04366739,NCT04354805 |
| Drug 122 | thalidomide | 2 | NCT04273529,NCT04273581 |
| Drug 123 | brequinar | 2 | NCT04425252,NCT04575038 |
| Drug 124 | eculizumab | 2 | NCT04346797,NCT04288713 |
| Drug 125 | valsartan | 2 | NCT04883528,NCT04335786 |
| Drug 126 | sildenafil | 2 | NCT04304313,NCT04489446 |
| Drug 127 | bemiparin | 2 | NCT04604327,NCT04420299 |
| Drug 128 | disulfiram | 2 | NCT04485130,NCT04594343 |
| Drug 129 | calcifediol | 2 | NCT04386850, NCT04366908 |
| Drug 130 | captopril | 2 | NCT04578236,NCT04355429 |
| Drug 131 | siltuximab | 2 | NCT04330638,NCT04329650 |
| Drug 132 | simvastatin | 2 | NCT04348695,NCT02735707 |
| Drug 133 | thymalfasin | 2 | NCT04428008,NCT04487444 |
| Drug 134 | selinexor | 2 | NCT04534725,NCT04349098 |
| Drug 135 | acetaminophen | 2 | NCT04673214,NCT04324606 |
| Drug 136 | enzalutamide | 2 | NCT04475601,NCT04456049 |
| Drug 137 | alteplase | 2 | NCT04357730,NCT04640194 |
| Drug 138 | budesonide | 2 | NCT04361474,NCT04355637 |
| Drug 139 | cetylpyridinium | 2 | NCT04584684,NCT04721457 |
| Drug 140 | iodine | 2 | NCT04473261,NCT04510402 |
| Drug 141 | clarithromycin | 2 | NCT04622891,NCT04398004 |
| Drug 142 | tacrolimus | 2 | NCT04341038,NCT04701528 |
| Drug 143 | human immunoglobulin g | 2 | NCT04500067,NCT04616001 |
| Drug 144 | leflunomide | 2 | NCT04361214,NCT04532372 |
| Drug 145 | interleukin-7 | 2 | NCT04407689,NCT04379076 |
| Drug 146 | linagliptin | 2 | NCT04542213,NCT04341935 |
| Drug 147 | mefloquine | 2 | NCT04847661,NCT04347031 |
| Drug 148 | folic acid | 2 | NCT04354428,NCT04631536 |
| Drug 149 | nitrogen | 2 | NCT04528771,NCT03331445 |
| Drug 150 | fondaparinux | 2 | NCT04359212,NCT04368377 |
| Drug 151 | estradiol | 2 | NCT04853069,NCT04359329 |
| Drug 152 | ofatumumab | 2 | NCT04878211,NCT04869358 |
| Drug 153 | fluoxetine | 2 | NCT04377308,NCT04780152 |
| Drug 154 | ledipasvir | 2 | NCT04530422,NCT04498936 |
| Drug 155 | progesterone | 2 | NCT04365127,NCT04865029 |
| Drug 156 | liothyronine | 2 | NCT04348513,NCT04725110 |
| Drug 157 | ketamine | 2 | NCT04365985,NCT04769297 |
| Drug 158 | plitidepsin | 2 | NCT04382066,NCT04784559 |
| Drug 159 | prasugrel | 2 | NCT04445623,NCT02735707 |
| Drug 160 | peginterferon lambda-1a | 2 | NCT04331899,NCT04354259 |
| Drug 161 | human interferon beta | 2 | NCT04521400,NCT04324463 |
| Drug 162 | ibuprofen | 2 | NCT04334629,NCT04382768 |
| Drug 163 | iloprost | 2 | NCT04420741,NCT04445246 |
| Drug 164 | inosine pranobex | 2 | NCT04360122,NCT04383717 |
| Drug 165 | conestat alfa | 1 | NCT04414631 |
| Drug 166 | decitabine | 1 | NCT04482621 |
| Drug 167 | dasatinib | 1 | NCT04830735 |
| Drug 168 | clindamycin | 1 | NCT04349410 |
| Drug 169 | clofazimine | 1 | NCT04465695 |
| Drug 170 | dexmedetomidine | 1 | NCT04358627 |
| Drug 171 | clavulanic acid | 1 | NCT04363060 |
| Drug 172 | citrulline | 1 | NCT04570384 |
| Drug 173 | cetirizine | 1 | NCT04836806 |
| Drug 174 | degarelix | 1 | NCT04397718 |
| Drug 175 | adalimumab | 1 | NCT04705844 |
| Drug 176 | dimethyl fumarate | 1 | NCT04381936 |
| Drug 177 | bardoxolone methyl | 1 | NCT04494646 |
| Drug 178 | adenosine | 1 | NCT04588441 |
| Drug 179 | alisporivir | 1 | NCT04608214 |
| Drug 180 | amoxicillin | 1 | NCT04363060 |
| Drug 181 | amlodipine | 1 | NCT04559074 |
| Drug 182 | ceftriaxone | 1 | NCT02735707 |
| Drug 183 | almitrine | 1 | NCT04357457 |
| Drug 184 | amiodarone | 1 | NCT04351763 |
| Drug 185 | alprostadil | 1 | NCT04536363 |
| Drug 186 | celecoxib | 1 | NCT04488081 |
| Drug 187 | diphenhydramine | 1 | NCT04456439 |
| Drug 188 | ceftaroline fosamil | 1 | NCT02735707 |
| Drug 189 | cefditoren | 1 | NCT04709172 |
| Drug 190 | theophylline | 1 | NCT04789499 |
| Drug 191 | secukinumab | 1 | NCT04403243 |
| Drug 192 | tamoxifen | 1 | NCT04389580 |
| Drug 193 | quetiapine | 1 | NCT04513314 |
| Drug 194 | quinine | 1 | NCT04553705 |
| Drug 195 | tenofovir alafenamide | 1 | NCT04405271 |
| Drug 196 | ramelteon | 1 | NCT04470297 |
| Drug 197 | ramipril | 1 | NCT04366050 |
| Drug 198 | tenecteplase | 1 | NCT04505592 |
| Drug 199 | tazobactam | 1 | NCT04394182 |
| Drug 200 | abatacept | 1 | NCT04593940 |
| Drug 201 | iodide | 1 | NCT04682873 |
| Drug 202 | omeprazole | 1 | NCT04333407 |
| Drug 203 | aprotinin | 1 | NCT04527133 |
| Drug 204 | bempegaldesleukin | 1 | NCT04646044 |
| Drug 205 | berberine | 1 | NCT04479202 |
| Drug 206 | bivalirudin | 1 | NCT04445935 |
| Drug 207 | brexanolone | 1 | NCT04537806 |
| Drug 208 | bucillamine | 1 | NCT04504734 |
| Drug 209 | calcium | 1 | NCT04379310 |
| Drug 210 | candesartan | 1 | NCT04351724 |
| Drug 211 | aprepitant | 1 | NCT04470622 |
| Drug 212 | ketotifen | 1 | NCT04435028 |
| Drug 213 | argatroban | 1 | NCT04406389 |
| Drug 214 | nitroglycerin | 1 | NCT04686760 |
| Drug 215 | epoprostenol | 1 | NCT04452669 |
| Drug 216 | masitinib | 1 | NCT04622865 |
| Drug 217 | eltrombopag | 1 | NCT04516837 |
| Drug 218 | medical cannabis | 1 | NCT03944447 |
| Drug 219 | edoxaban | 1 | NCT04516941 |
| Drug 220 | melphalan | 1 | NCT04380376 |
| Drug 221 | furosemide | 1 | NCT04588792 |
| Drug 222 | isoflurane | 1 | NCT04415060 |
| Drug 223 | itraconazole | 1 | NCT04577378 |
| Drug 224 | nicotinamide | 1 | NCT04910230 |
| Drug 225 | losmapimod | 1 | NCT04511819 |
| Drug 226 | methotrexate | 1 | NCT04352465 |
| Drug 227 | nicorandil | 1 | NCT04631536 |
| Drug 228 | modafinil | 1 | NCT04751227 |
| Drug 229 | nebivolol | 1 | NCT04631536 |
| Drug 230 | moxifloxacin | 1 | NCT02735707 |
| Drug 231 | propranolol | 1 | NCT04467086 |
| Drug 232 | montmorillonite | 1 | NCT04479202 |
| Drug 233 | mometasone furoate | 1 | NCT04484493 |
| Drug 234 | molgramostim | 1 | NCT04569877 |
| Drug 235 | lucinactant | 1 | NCT04389671 |
| Drug 236 | escin | 1 | NCT04322344 |
| Drug 237 | arginine | 1 | NCT04631536 |
| Drug 238 | lenalidomide | 1 | NCT04361643 |
| Drug 239 | aspartic acid | 1 | NCT04703608 |
| Drug 240 | atovaquone | 1 | NCT04339426 |
| Drug 241 | diosmin | 1 | NCT04452799 |
| Drug 242 | dronabinol | 1 | NCT04615949 |
| Drug 243 | omega-3 fatty acids | 1 | NCT04495816 |
| Drug 244 | interferon alfa | 1 | NCT04534725 |
| Drug 245 | dutasteride | 1 | NCT04729491 |
| Drug 246 | lactose | 1 | NCT04894617 |
| Drug 247 | lanadelumab | 1 | NCT04590586 |
| Drug 248 | indomethacin | 1 | NCT04344457 |
| Drug 249 | etoposide | 1 | NCT04356690 |
| Drug 250 | icosapent | 1 | NCT04335032 |
| Drug 251 | icatibant | 1 | NCT04488081 |
| Drug 252 | levofloxacin | 1 | NCT02735707 |
| Drug 253 | ibudilast | 1 | NCT04429555 |
| Drug 254 | hypochlorous acid | 1 | NCT04684550 |
| Drug 255 | hypochlorite | 1 | NCT04721457 |
| Drug 256 | glatiramer | 1 | NCT04878211 |
| Drug 257 | licorice | 1 | NCT04553705 |
| Drug 258 | isavuconazonium | 1 | NCT04707703 |
| Drug 259 | thimerosal | 1 | NCT04522830 |
| Drug 260 | trimetazidine | 1 | NCT04760821 |
| Drug 261 | trastuzumab | 1 | NCT04395508 |
| Drug 262 | sorbitol | 1 | NCT04573153 |
| Drug 263 | recombinant human thrombopoietin | 1 | NCT04516837 |
| Drug 264 | sulfur hexafluoride | 1 | NCT04640038 |
| Drug 265 | sulodexide | 1 | NCT04483830 |
| Drug 266 | tirofiban | 1 | NCT04368377 |
| Drug 267 | tetracycline | 1 | NCT04716426 |
| Drug 268 | piperacillin | 1 | NCT04394182 |
| Drug 269 | verapamil | 1 | NCT04351763 |
| Drug 270 | tramadol | 1 | NCT04454307 |
| Drug 271 | pertuzumab | 1 | NCT04395508 |
| Drug 272 | voclosporin | 1 | NCT04701528 |
| Drug 273 | pentaglobin | 1 | NCT04351724 |
| Drug 274 | pacritinib | 1 | NCT04404361 |
| Drug 275 | xylitol | 1 | NCT04854486 |
| Drug 276 | zanubrutinib | 1 | NCT04382586 |
| Drug 277 | ozanimod | 1 | NCT04405102 |
| Drug 278 | valproic acid | 1 | NCT04513314 |
| Drug 279 | vitamin a | 1 | NCT04900415 |
| Drug 280 | propofol | 1 | NCT04359862 |
| Drug 281 | prazosin | 1 | NCT04365257 |
| Drug 282 | ulinastatin | 1 | NCT04393311 |
| Drug 283 | poractant alfa | 1 | NCT04384731 |
| Drug 284 | regadenoson | 1 | NCT04606069 |
| Drug 285 | vitamin e | 1 | NCT04570254 |
| Drug 286 | semaglutide | 1 | NCT04615871 |
| Drug 287 | pyridostigmine | 1 | NCT04343963 |
| Drug 288 | salbutamol | 1 | NCT04681079 |
| Drug 289 | selenium | 1 | NCT04869579 |
| Drug 290 | resveratrol | 1 | NCT04799743 |
| Drug 291 | tafenoquine | 1 | NCT04533347 |
| Drug 292 | sacubitril | 1 | NCT04883528 |
| Drug 293 | rosuvastatin | 1 | NCT04359095 |
| Drug 294 | sirukumab | 1 | NCT04380961 |
| Drug 295 | ticagrelor | 1 | NCT02735707 |
| Drug 296 | sitagliptin | 1 | NCT04365517 |
| Drug 297 | remdesivir | 50 | NCT04582266,NCT04492475,NCT04401579,NCT04280705,NCT04583969,NCT04640168,NCT04583956,NCT04365725,NCT04330690,NCT04351724,NCT04292899,NCT04488081,NCT04539262,NCT04745351,NCT04292730,NCT04431453,NCT04871633,NCT04593940,NCT04713176,NCT04784559,NCT04409262,NCT04678739,NCT04843761,NCT04501978,NCT04853901,NCT04647695,NCT04647669,NCT04391309,NCT04779047,NCT04669990,NCT04694612,NCT04738045,NCT04832880,NCT04596839,NCT04854837,NCT04575064,NCT04693026,NCT04847622,NCT04349410,NCT04492501,NCT04323761,NCT04302766,NCT04315948,NCT04345419,NCT04321616,NCT04560231,NCT04546581,NCT04865237,NCT04728880,NCT04727775 |
| Drug 298 | povidone-iodine | 13 | NCT04603794,NCT04721457, NCT04410159,NCT04347954,NCT04371965,NCT04449965,NCT04478019,NCT04364802,NCT04549376,NCT04446104,NCT04517188,NCT04393792, NCT04872686 |
| Drug 299 | niclosamide | 10 | NCT04603924,NCT04399356,NCT04858425,NCT04542434,NCT04870333,NCT04558021,NCT04753619,NCT04436458,NCT04750759,NCT04644705 |
| Drug 300 | azd7442 | 6 | NCT04723394,NCT04625972,NCT04625725,NCT04501978,NCT04518410,NCT04315948 |
| Drug 301 | proxalutamide | 5 | NCT04853134,NCT04728802,NCT04853927,NCT04446429,NCT04870606 |
| Drug 302 | leronlimab | 5 | NCT04901676,NCT04901689,NCT04678830,NCT04343651,NCT04347239 |
| Drug 303 | bamlanivimab | 5 | NCT04701658,NCT04656691,NCT04603651,NCT04518410,NCT04748588 |
| Drug 304 | mavrilimumab | 5 | NCT04447469,NCT04463004,NCT04492514,NCT04399980,NCT04397497 |
| Drug 305 | eidd-2801 | 4 | NCT04746183,NCT04405570,NCT04405739,NCT04392219 |
| Drug 306 | vir-7831 | 4 | NCT04634409,NCT04545060,NCT04746183,NCT04501978 |
| Drug 307 | aviptadil | 4 | NCT04844580,NCT04843761,NCT04311697,NCT04536350 |
| Drug 308 | lenzilumab | 3 | NCT04583969,NCT04351152,NCT04534725 |
| Drug 309 | covid-19 convalescent plasma | 3 | NCT04428021,NCT04345991,NCT04395170 |
| Drug 310 | isoquercetin | 3 | NCT04536090,NCT04733651,NCT04622865 |
| Drug 311 | opaganib | 3 | NCT04467840,NCT04435106,NCT04414618 |
| Drug 312 | cenicriviroc | 3 | NCT04488081,NCT04593940,NCT04500418 |
| Drug 313 | icosapent ethyl | 3 | NCT04412018,NCT04460651,NCT04505098 |
| Drug 314 | adipose-derived mesenchymal stem cells: autologous or allogeneic origins | 3 | NCT04527224, NCT04348435,NCT04349631 |
| Drug 315 | fisetin | 3 | NCT04476953,NCT04771611,NCT04537299 |
| Drug 316 | mycobacterium w. vaccine | 3 | NCT04353518,NCT04358809,NCT04347174 |
| Drug 317 | remestemcel-l | 3 | NCT04366830,NCT04456439,NCT04371393 |
| Drug 318 | olokizumab | 2 | NCT04452474,NCT04380519 |
| Drug 319 | ebselen | 2 | NCT04483973,NCT04484025 |
| Drug 320 | silmitasertib | 2 | NCT04663737,NCT04668209 |
| Drug 321 | acetate | 2 | NCT04621149,NCT04878211 |
| Drug 322 | chlorine dioxide | 2 | NCT04621149,NCT04343742 |
| Drug 323 | acebilustat | 2 | NCT04662086,NCT04662060 |
| Drug 324 | ec-18 | 2 | NCT04500132,NCT04569227 |
| Drug 325 | angiotensin 1-7 | 2 | NCT04605887,NCT04332666 |
| Drug 326 | clevudine | 2 | NCT04347915,NCT04891302 |
| Drug 327 | ravulizumab | 2 | NCT04390464,NCT04570397 |
| Drug 328 | ino-4800 | 2 | NCT04642638,NCT04336410 |
| Drug 329 | iota-carrageenan | 2 | NCT04521322,NCT04701710 |
| Drug 330 | efineptakin alfa | 2 | NCT04501796,NCT04498325 |
| Drug 331 | zinc sulfate | 2 | NCT04370782,NCT04621461 |
| Drug 332 | nangibotide | 1 | NCT04429334 |
| Drug 333 | fx06 | 1 | NCT04618042 |
| Drug 334 | ensifentrine | 1 | NCT04527471 |
| Drug 335 | glenzocimab | 1 | NCT04659109 |
| Drug 336 | itolizumab | 1 | NCT04475588 |
| Drug 337 | gs-441524 | 1 | NCT04859244 |
| Drug 338 | eritoran | 1 | NCT02735707 |
| Drug 339 | estetrol | 1 | NCT04801836 |
| Drug 340 | abivertinib | 1 | NCT04440007 |
| Drug 341 | gimsilumab | 1 | NCT04351243 |
| Drug 342 | metenkefalin | 1 | NCT04374032 |
| Drug 343 | nicotinamide riboside | 1 | NCT04818216 |
| Drug 344 | estradiol cypionate | 1 | NCT04865029 |
| Drug 345 | exebacase | 1 | NCT04597242 |
| Drug 346 | fosmanogepix | 1 | NCT04240886 |
| Drug 347 | monalizumab | 1 | NCT04333914 |
| Drug 348 | narsoplimab | 1 | NCT04488081 |
| Drug 349 | ft516 | 1 | NCT04363346 |
| Drug 350 | emricasan | 1 | NCT04803227 |
| Drug 351 | mrx-4dp0004 | 1 | NCT04363372 |
| Drug 352 | human umbilical cord mesenchymal stem cells | 1 | NCT04390139 |
| Drug 353 | protoporphyrin | 1 | NCT04371822 |
| Drug 354 | ltx-109 | 1 | NCT04854928 |
| Drug 355 | peppermint | 1 | NCT04864925 |
| Drug 356 | desidustat | 1 | NCT04463602 |
| Drug 357 | pf-06650833 | 1 | NCT04575610 |
| Drug 358 | dapansutrile | 1 | NCT04540120 |
| Drug 359 | dalcetrapib | 1 | NCT04676867 |
| Drug 360 | cumin | 1 | NCT04347382 |
| Drug 361 | piclidenoson | 1 | NCT04333472 |
| Drug 362 | vb-201 | 1 | NCT04733833 |
| Drug 363 | primaquine | 1 | NCT04349410 |
| Drug 364 | tradipitant | 1 | NCT04326426 |
| Drug 365 | recombinant human angiotensin-converting enzyme 2 | 1 | NCT04335136 |
| Drug 366 | recombinant human plasma gelsolin | 1 | NCT04358406 |
| Drug 367 | brilacidin | 1 | NCT04784897 |
| Drug 368 | brensocatib | 1 | NCT04817332 |
| Drug 369 | bgb-dxp593 | 1 | NCT04551898 |
| Drug 370 | remimazolam | 1 | NCT04611425 |
| Drug 371 | reparixin | 1 | NCT04878055 |
| Drug 372 | rnapc2 | 1 | NCT04655586 |
| Drug 373 | senicapoc | 1 | NCT04594668 |
| Drug 374 | tempol | 1 | NCT04729595 |
| Drug 375 | recombinant bacterial ace2 receptors-like enzyme | 1 | NCT04375046 |
| Drug 376 | razuprotafib | 1 | NCT04511650 |
| Drug 377 | ptc299 | 1 | NCT04439071 |
| Drug 378 | tetrandrine | 1 | NCT04308317 |
| Drug 379 | bemcentinib | 1 | NCT04890509 |
| Drug 380 | azvudine | 1 | NCT04668235 |
| Drug 381 | levilimab | 1 | NCT04397562 |
| Drug 382 | anti-sars-cov-2 immunoglobulin | 1 | NCT04546581 |
| Drug 383 | umifenovir | 1 | NCT04350684 |
| Drug 384 | sars-cov-2 mrna vaccine | 1 | NCT04843774 |
| Drug 385 | tridecactide | 1 | NCT04374032 |
| Drug 386 | triazavirin | 1 | NCT04581915 |
| Drug 387 | azd1222 | 1 | NCT04568031 |
| Drug 388 | avdoralimab | 1 | NCT04333914 |
| Drug 389 | avasopasem manganese | 1 | NCT04555096 |
| Drug 390 | treamid | 1 | NCT04527354 |
| Drug 391 | at-001 | 1 | NCT04365699 |
| Drug 392 | asunercept | 1 | NCT04351724 |
| Drug 393 | upamostat | 1 | NCT04723537 |
| Drug 394 | vadadustat | 1 | NCT04478071 |
| Drug 395 | apilimod | 1 | NCT04446377 |
| Drug 396 | apabetalone | 1 | NCT04894266 |
| Drug 397 | azoximer bromide | 1 | NCT04381377 |
| Drug 398 | antroquinonol | 1 | NCT04523181 |
| Drug 399 | anti-sars-cov-2 igy | 1 | NCT04567810 |
| Drug 400 | anti-sars-cov-2 equine immunoglobulin fragments | 1 | NCT04514302 |
| Drug 401 | amy-101 | 1 | NCT04395456 |
| Drug 402 | zilucoplan | 1 | NCT04590586 |
| Drug 403 | alvelestat | 1 | NCT04539795 |
| Drug 404 | xav-19 | 1 | NCT04453384 |
| Drug 405 | ak119 | 1 | NCT04516564 |
| Drug 406 | zotatifin | 1 | NCT04632381 |

**Supplementary Table 2**. Drugs in Anatomical Therapeutic Chemical (ATC) classes

| ATC Classes | # of drugs | Drug Name |
| --- | --- | --- |
| Alimentary tract and metabolism | 29 | acetylsalicylic acid, calcifediol, hydrogen peroxide, cholecalciferol, ascorbic acid, sodium chloride, hydrocortisone, chlorhexidine, dexamethasone, dapagliflozin, nicotinamide, prednisolone, tetracycline, zinc sulfate, pioglitazone, linagliptin, doxycycline, sitagliptin, semaglutide, aprepitant, omeprazole, prednisone, famotidine, budesonide, metformin, vitamin d, sorbitol, calcium, dronabinol |
| Blood and blood forming organs | 38 | heparin, acetylsalicylic acid, sodium bicarbonate, antithrombin iii human, cetylpyridinium, sodium chloride, chlorhexidine, conestat alfa, fostamatinib, tenecteplase, epoprostenol, fondaparinux, dipyridamole, lanadelumab, eltrombopag, clopidogrel, rivaroxaban, defibrotide, bivalirudin, argatroban, dalteparin, tinzaparin, folic acid, sulodexide, enoxaparin, ticagrelor, bemiparin, alteplase, tirofiban, aprotinin, icatibant, prasugrel, sorbitol, edoxaban, camostat, iloprost, apixaban, dextrose, unspecified form |
| Cardiovascular system | 34 | heparin, alprostadil, pentoxifylline, hydrocortisone, angiotensin ii, spironolactone, trimetazidine, dexamethasone, atorvastatin, prednisolone, rosuvastatin, indomethacin, ambrisentan, regadenoson, propranolol, simvastatin, telmisartan, candesartan, amlodipine, nicorandil, furosemide, amiodarone, lidocaine, valsartan, ibuprofen, adenosine, verapamil, captopril, nebivolol, prazosin, losartan, ramipril, diosmin, zinc |
| Dermatologicals | 19 | tretinoin, methylprednisolone, hydrogen peroxide, cetylpyridinium, povidone-iodine, diphenhydramine, hydrocortisone, chlorhexidine, dexamethasone, prednisolone, isotretinoin, tetracycline, clindamycin, ivermectin, tacrolimus, budesonide, lidocaine, ethanol, iodine |
| Genitourinary system and sex hormones | 9 | alprostadil, ascorbic acid, povidone-iodine, progesterone, clindamycin, dutasteride, sildenafil, ibuprofen, estradiol |
| Systemic hormonal preparations | 6 | methylprednisolone, hydrocortisone, dexamethasone, prednisolone, prednisone, liothyronine |
| Antiinfectives for systemic use | 30 | tenofovir alafenamide, tenofovir disoproxil, clarithromycin, emtricitabine, inosine pranobex, azithromycin, itraconazole, moxifloxacin, tetracycline, piperacillin, levofloxacin, ceftaroline fosamil, clindamycin, amoxicillin, daclatasvir, oseltamivir, clofazimine, ceftriaxone, doxycycline, cefditoren, sofosbuvir, atazanavir, umifenovir, tazobactam, ribavirin, darunavir, ritonavir, maraviroc, lopinavir, clevudine |
| Antineoplastic and immunomodulating agents | 51 | tretinoin, dasatinib, interferon beta-1a, interferon beta-1b, dimethyl fumarate, cyclosporine, bicalutamide, methotrexate, lenalidomide, molgramostim, sargramostim, enzalutamide, tofacitinib, bcg vaccine, thalidomide, plitidepsin, voclosporin, trastuzumab, tocilizumab, canakinumab, pirfenidone, leflunomide, bevacizumab, secukinumab, ruxolitinib, baricitinib, tacrolimus, nintedanib, adalimumab, ofatumumab, siltuximab, apremilast, eculizumab, pertuzumab, infliximab, decitabine, tamoxifen, ibrutinib, sirukumab, etoposide, abatacept, degarelix, masitinib, celecoxib, sirolimus, nivolumab, melphalan, sarilumab, imatinib, ozanimod, anakinra |
| Musculo-sketal system | 5 | indomethacin, bucillamine, colchicine, ibuprofen, celecoxib |
| Nervous system | 23 | acetylsalicylic acid, dexmedetomidine, chlorpromazine, pyridostigmine, fluvoxamine, sevoflurane, acetaminophen, cannabidiol, disulfiram, naltrexone, quetiapine, fluoxetine, isoflurane, amantadine, ramelteon, lidocaine, melatonin, modafinil, valproic acid, nicotine, propofol, tramadol, ketamine |
| Antiparasitic products | 13 | hydroxychloroquine, nitazoxanide, artemisinin, niclosamide, chloroquine, disulfiram, ivermectin, levamisole, mefloquine, primaquine, artesunate, atovaquone, quinine |
| Respiratory system | 22 | acetylcysteine, cetylpyridinium, povidone-iodine, diphenhydramine, cyproheptadine, chlorhexidine, dexamethasone, dornase alfa, theophylline, prednisolone, nitric oxide, ciclesonide, montelukast, cetirizine, bromhexine, budesonide, ketotifen, ibudilast, lidocaine, ibuprofen, salbutamol, almitrine |
| Sensory organs | 19 | heparin, hydrogen peroxide, acetylcysteine, ascorbic acid, povidone-iodine, hydrocortisone, chlorhexidine, cyclosporine, dexamethasone, azithromycin, moxifloxacin, prednisolone, tetracycline, indomethacin, levofloxacin, ketotifen, lidocaine, alteplase, sirolimus |
| Various | 10 | sulfur hexafluoride, acetylcysteine, methylene blue, deferoxamine, cobicistat, sorbitol, nitrogen, ethanol, dextrose, unspecified form, oxygen |

**Supplementary Table 3**. System Organ Class names and corresponding abbreviations

| System Organ Class Name | System Organ Class Abbreviation |
| --- | --- |
| Blood and lymphatic system disorders | blood |
| Cardiac disorders | card |
| Congenital, familial and genetic disorders | cong |
| Ear and labyrinth disorders | ear |
| Endocrine disorders | endo |
| Eye disorders | eye |
| Gastrointestinal disorders | gastr |
| General disorders and administration site conditions | genrl |
| Hepatobiliary disorders | hepat |
| Immune system disorders | immun |
| Infections and infestations | infec |
| Injury, poisoning and procedural complications | inj&p |
| Investigations | inv |
| Metabolism and nutrition disorders | metab |
| Musculoskeletal and connective tissue disorders | musc |
| Neoplasms benign, malignant and unspecified (incl cysts and polyps) | neopl |
| Nervous system disorders | nerv |
| Pregnancy, puerperium and perinatal conditions | preg |
| Product issues | prod |
| Psychiatric disorders | psych |
| Renal and urinary disorders | renal |
| Reproductive system and breast disorders | repro |
| Respiratory, thoracic and mediastinal disorders | resp |
| Skin and subcutaneous tissue disorders | skin |
| Social circumstances | socci |
| Surgical and medical procedures | surg |
| Vascular disorders | vasc |
